# Development and Validation of the Isotretinoin Hesitancy Scale

**DOI:** 10.1192/j.eurpsy.2024.1019

**Published:** 2024-08-27

**Authors:** E. Agaoglu, I. G. Yilmaz-Karaman, C. Yastibas-Kacar, H. Kaya-Erdogan, T. Mutlu, E. Acer

**Affiliations:** ^1^Dermatology; ^2^Psychiatry, Eskişehir Osmangazi University, Eskişehir; ^3^Psychology, Adana Alparslan Türkeş Science and Technology University, Adana, Türkiye

## Abstract

**Introduction:**

Isotretinoin is an effective treatment for acne vulgaris; however, many patients experience anxiety while deciding to get it. Isotretinoin, indeed, has significant adverse effects. On the other hand, effective treatment of acne vulgaris may reduce dermatological and psychiatric complications.

**Objectives:**

The present study aims to develop and validate the Isotretionin Hesitancy Scale to measure the patients’ drawbacks to the treatment.

**Methods:**

The specialists, including dermatologists and mental health professionals, determined an item pool of 30 items. Before the data collection, all items were checked by the researchers in terms of clarity and acceptability. Thus, the eight items were removed from the questionnaire due to having similar meanings, measuring the facts about treatments that are not the study’s objective, and containing unclear statements. The final version of the questionnaire, which consists of 22 items, was applied to the participants.

**Results:**

One hundred patients with acne vulgaris were recruited. Among the participants, 72% were women, and the mean age was 22.72. Most patients’ acne severity was group 2 (40%) and group 3 (36%). Three items were removed because of having low item-total score correlations. Five items were removed in factor analysis because of low factor loading or cross-loading. Exploratory factor analysis results of the scale are presented in Table 1.

**Table 1.** Exploratory Factor Analysis Results of the Scale

	Factor 1 (min-max)	Factor 2 (min-max)	Factor 3 (min-max)
Isotretinoin treatment can lead to dryness of lips, nose, and eyes. Isotretinoin treatment may have many side effects. Isotretinoin treatment may cause damage to the liver. Side effects of isotretinoin treatment may affect my daily life. Isotretinoin treatment may cause depression. Isotretinoin treatment may cause elevation of cholesterol level.	0.516-0.842		
Isotretinoin treatment may cause infertility in men. Isotretinoin treatment may cause infertility in women. Isotretinoin treatment may prevent height gain. In case of pregnancy, isotretinoin treatment may cause congenital defects in the baby.		0.425-0.945	
I’m afraid of using isotretinoin for a long period. I stop the isotretinoin treatment as soon as possible. I will wait as long as I can before using isotretinoin treatment. I need more reassurance about isotretinoin treatment.			0.569-0.890

The Cronbach alpha score of the final form of the scale was found to be .81, the internal consistency of the first factor (hesitancy related to reversible adverse effects) was calculated as .79, the second factor (hesitancy related to irreversible adverse effects) was calculated as .78, and the final factor (isotretinoin-related anxiety) was found to be .72.

**Conclusions:**

The Isotretionin Hesitancy Scale is valid and reliable among patients with acne vulgaris.

**Disclosure of Interest:**

None Declared

